# Upregulation of Hemoglobin Expression by Oxidative Stress in Hepatocytes and Its Implication in Nonalcoholic Steatohepatitis

**DOI:** 10.1371/journal.pone.0024363

**Published:** 2011-09-12

**Authors:** Wensheng Liu, Susan S. Baker, Robert D. Baker, Norma J. Nowak, Lixin Zhu

**Affiliations:** 1 Digestive Diseases and Nutrition Center, Department of Pediatrics, The State University of New York at Buffalo, Buffalo, New York, United States of America; 2 Department of Biochemistry and the New York State Center of Excellence in Bioinformatics and Life Sciences, The State University of New York at Buffalo, Buffalo, New York, United States of America; 3 Microarray and Genomics Facility, Roswell Park Cancer Institute, Buffalo, New York, United States of America; Chinese University of Hong Kong, Hong Kong

## Abstract

Recent studies revealed that hemoglobin is expressed in some non-erythrocytes and it suppresses oxidative stress when overexpressed. Oxidative stress plays a critical role in the pathogenesis of non-alcoholic steatohepatitis (NASH). This study was designed to investigate whether hemoglobin is expressed in hepatocytes and how it is related to oxidative stress in NASH patients. Analysis of microarray gene expression data revealed a significant increase in the expression of hemoglobin alpha (HBA1) and beta (HBB) in liver biopsies from NASH patients. Increased hemoglobin expression in NASH was validated by quantitative real time PCR. However, the expression of hematopoietic transcriptional factors and erythrocyte specific marker genes were not increased, indicating that increased hemoglobin expression in NASH was not from erythropoiesis, but could result from increased expression in hepatocytes. Immunofluorescence staining demonstrated positive HBA1 and HBB expression in the hepatocytes of NASH livers. Hemoglobin expression was also observed in human hepatocellular carcinoma HepG2 cell line. Furthermore, treatment with hydrogen peroxide, a known oxidative stress inducer, increased HBA1 and HBB expression in HepG2 and HEK293 cells. Importantly, hemoglobin overexpression suppressed oxidative stress in HepG2 cells. We concluded that hemoglobin is expressed by hepatocytes and oxidative stress upregulates its expression. Suppression of oxidative stress by hemoglobin could be a mechanism to protect hepatocytes from oxidative damage in NASH.

## Introduction

Nonalcoholic fatty liver disease (NAFLD) is a chronic liver disease closely associated with obesity and insulin resistance. With the increasing prevalence of obesity worldwide, NAFLD is becoming one of the most common liver diseases in adults and children. The hepatic lesions of NAFLD range from simple steatosis to nonalcoholic steatohepatitis (NASH), and to cirrhosis [1]–[3]. NASH is characterized by macrosteatosis, hepatocyte ballooning, and mixed lobular inflammation [4]–[8]. Although the pathogenesis of NASH is not fully understood, several lines of studies indicate that oxidative stress, an imbalance between the production of reactive oxygen species and antioxidants, plays a central role in the progression from simple steatosis to NASH [9]–[13]. Increased oxidative stress and decreased antioxidant enzymes such as glutathione peroxidase and superoxide dismutase have been reported in NASH [14]–[16]. In some clinical studies antioxidants are shown to have beneficial effects on the livers of patients with NASH [17], [18]. At present, liver biopsy remains the golden standard for NASH diagnosis. Several promising serum biomarkers such as cytokeratin 18 need to be validated for non invasive diagnosis for NASH in large scale clinical trials [19]. A recent proteomic study has shown that free hemoglobin subunits α and β in serum are significantly increased in NASH patients as compared to those with steatosis [20], [21]The source of free serum hemoglobin in serum is unknown, but presumed to result from oxidative stress-induced hemolysis.

Hemoglobin is a predominant component in erythrocytes and functions as an oxygen transporter in blood. The major hemoglobin form in adults is a tetramer consisting of two α- and β-subunits (α2 β2), each of which contains a heme group [22], [23]. Recently hemoglobin expression has been reported in non erythrocytes including neurons [24]–[26], retinal cells [27], [28], alveolar cells [29]–[31], mesangial cells of the kidney [32], and macrophages [33]. In alveolar cells, hemoglobin expression is induced by hypoxia [29]. Hemoglobin overexpression in murine MN9D [24] and SV40-MES13 [32] cells affects expression of various genes involved in O2 homeostasis or suppresses oxidative stress, respectively. The function and regulation of non-erythrocyte hemoglobin is not fully understood.

We report that hemoglobin is expressed in hepatocytes and is increased in NASH. Oxidative stress upregulates hemoglobin expression and hemoglobin overexpression suppresses oxidative stress in HepG2 cells. These findings suggest that hemoglobin plays a protective role in NASH.

## Results

### Elevated hemoglobin gene expression in NASH liver biopsies

**Figure 1:**
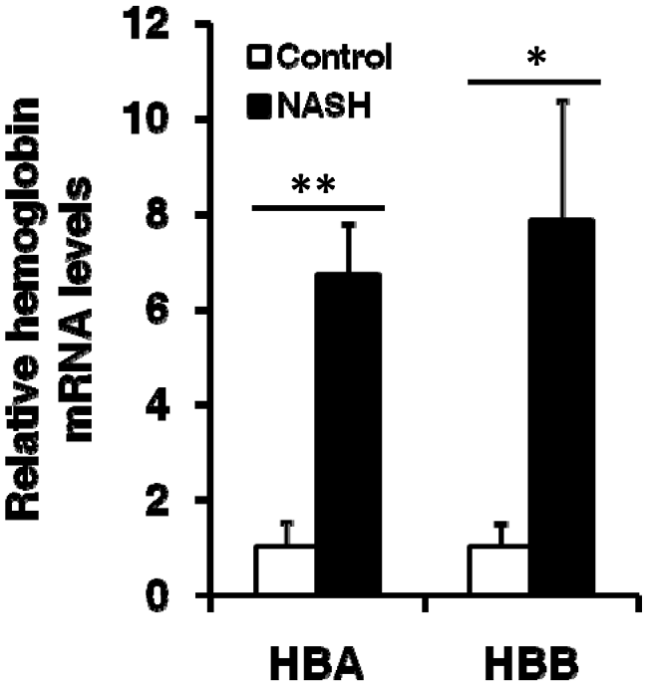
Increased expression of HBA1 and HBB in NASH livers. Quantitative real time PCR was performed with the same NASH (n = 12) and control (n = 5) samples used for microarray analysis. GAPDH was used as an endogenous control. Hemoglobin levels in controls were normalized to 1. Data were expressed as Mean ± SD. * and ** represent *P*<.05 and *P*<.01, respectively.

To identify differentially expressed genes in NASH, we analyzed a recently published microarray dataset including 12 NASH and 5 control samples. Compared to non-NASH controls, NASH samples showed a significant increase in the expression of HBA1 and HBB genes (Table 1). However, the expression of transcription factors for erythroid differentiation including GATA1, NFE2, KLF1, and TAL1 did not increase. In addition, the expression of other hemoglobin genes (HBD, HBE1, HBG2, HBQ1, and HBZ) and erythrocyte specific marker genes [34], [35] such as SPTA, SPTB, GYPA, and ALAS2 did not show a significant increase, suggesting that increased HBA1 and HBB expression in NASH did not result from erythropoiesis, but from a different mechanism. Increased HBA1 and HBB expression in NASH livers was validated by qRT-PCR. A 6.7 and 7.9 fold increase in the relative copy numbers were detected in NASH livers for HBA1 and HBB, respectively (Fig. 1).

**Table 1:** Increased HBA1 and HBB expression in NASH.

Gene Name	Gene Symbol	NASH	Control	NASH/Control	*P* Value
Hemoglobin family					
Hemoglobin, alpha 1	HBA1	112.3	29.83	3.77	0.023
Hemoglobin, beta	HBB	151.2	41.63	3.63	0.030
Hemoglobin, delta	HBD	0.28	2.59	0.11	0.141
Hemoglobin, epsilon 1	HBE1	7.78	8.64	0.90	0.367
Hemoglobin, gamma G	HBG2	0.30	0.46	0.65	0.099
Hemoglobin, theta 1	HBQ1	0.48	0.31	1.57	0.269
Hemoglobin, zeta	HBZ	1.12	0.82	1.37	0.084
Transcription factors for erythroid differentiation					
GATA binding protein 1 (globin transcription factor 1)	GATA1	8.69	18.99	0.46	0.000
Nuclear factor (erythroid-derived 2)	NFE2	0.27	0.46	0.59	0.109
Kruppel-like factor 1 (erythroid)	KLF1/EKLF	0.27	0.39	0.70	0.335
T-cell acute lymphocytic leukemia 1	TAL1/SCL	6.37	7.78	0.82	0.035
Erythroid cell markers					
Aminolevulinate, delta-, synthase 2	ALAS2	0.55	0.69	0.80	0.214
Glycophorin A	GYPA	11.97	21.95	0.55	0.000
Glycophorin B	GYPB	0.17	0.19	0.86	0.830
Spectrin, beta, erythrocytic	SPTB	0.39	0.33	1.19	0.703
Erythroid-associated factor	ERAF	22.09	19.47	1.13	0.389

### Detection of hemoglobin proteins in NASH liver biopsies

**Figure 2:**
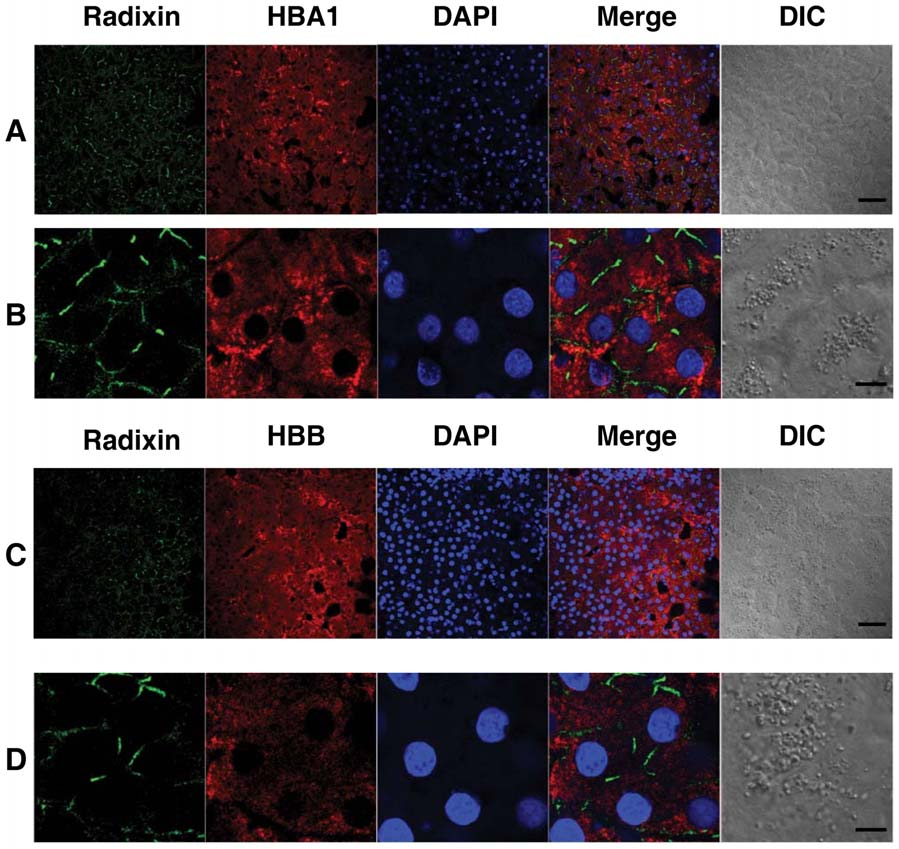
HBA1 and HBB expression in the hepatocytes of NASH livers. Immunofluorescence staining was performed on cryosections of NASH liver biopsies. Radixin is expressed in liver bile canaliculi. Radixin staining was used to identify hepatocytes. Images were recorded with an LSM510 beta laser scanning microscope (Zeiss). Cytoplasmic staining of HBA1 (**A**&**B**) and HBB (**C**&**D**) in hepatocytes of NASH livers were detected, respectively. Bars in A&C: 50 µm, bars in B&D: 10 µm.

Immunofluorescence (IF) staining of the cryosections from NASH biopsies were performed to examine the cellular distribution of HBA1 and HBB. As shown in Fig. 2, NASH liver biopsies exhibited positive signals for HBA1 (Fig. 2A&B) and HBB (Fig. 2C&D). Higher amplifications indicated that HBA1 and HBB proteins were expressed in the cytoplasm of the hepatocytes (Fig 2B &D). Radixin, specifically expressed in liver bile canaliculi [36], was used to identify hepatocytes.

### Detection of hemoglobin expression in HepG2 cells

**Figure 3:**
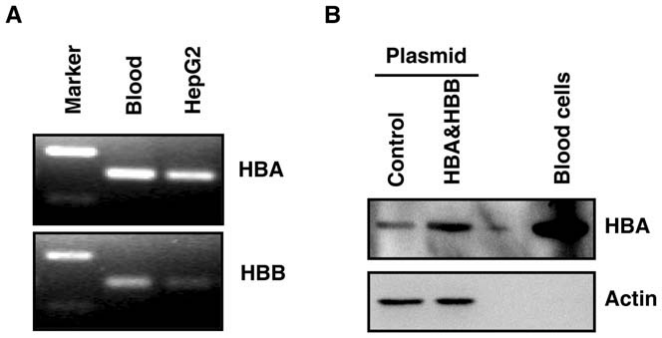
Expression of HBA1 and HBB in HepG2 cells. **A**, HBA1 and HBB mRNA were detected by reverse transcription PCR. PCR products were separated on 2% agrose gel and visualized with ethidium bromide. PCR products were further confirmed by sequencing. **B**, HBA1 protein was detected by Western blot analysis. Whole cell lysates from HepG2 cells transfected with control or HBA&HBB expression plasmid were analyzed for HBA1 protein. Blood cells were used as the positive control. Beta actin was probed as a loading control.

To further confirm that hemoglobin proteins were expressed in hepatocytes, expression of HBA1 and HBB was examined in the HepG2 cell line. First, HBA1 and HBB mRNA expression in HepG2 cells was examined using reverse transcription PCR. RNA extracted from blood cells was used as a positive control. HBA1 and HBB mRNA were detected in HepG2 cells (Fig. 3A). The PCR products were sequenced and exhibited 100% match with HBA1 (NM_000558) and HBB (NM_000518) mRNA sequences. Consistent with a previous study in alveolar cells [30], qRT-PCR showed that HBA1 expression is about 17 folds higher than HBB (Data not shown). Next, western blotting was performed to examine the expression of hemoglobin proteins in HepG2 cells. HBA1 proteins were detected in HepG2 cells (Fig. 3B). The HBA1 band (about 17 Kd) appeared at the same molecular weight as that from blood cells. Moreover, HBA1 signal was increased when HepG2 cells were transfected with HBA1 expression plasmid pIRES/HBA&HBB, demonstrating the HBA1 band detected in HepG2 cells was HBA1 specific. However, we were unable to detect HBB protein in HepG2 cells, probably because of the low expression level as suggested by RT-PCR.

### Upregulation of hemoglobin expression by oxidative stress

**Figure 4:**
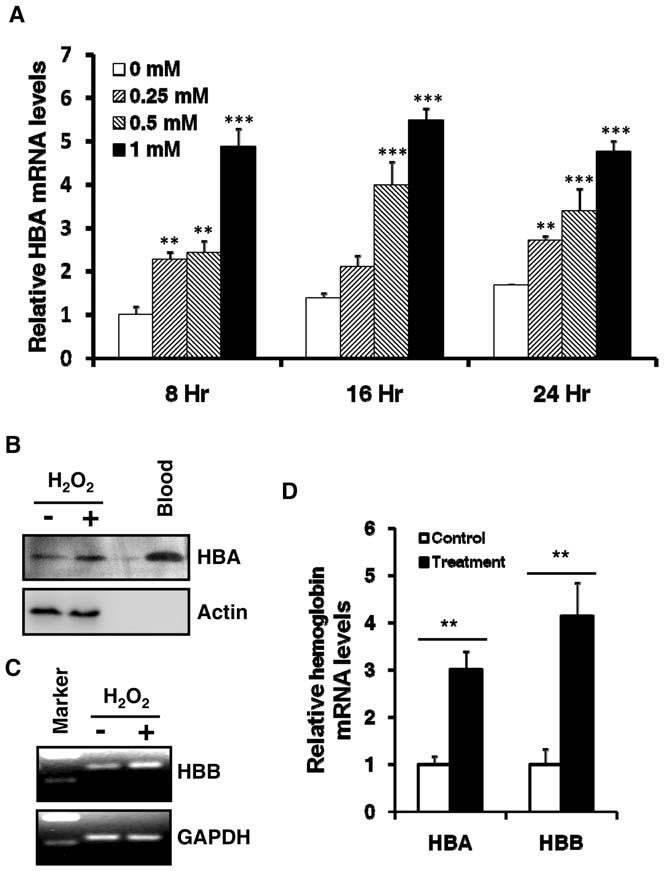
Upregulation of hemoglobin expression by oxidative stress. **A**, Induction of hemoglobin expression by H_2_O_2_ in a dose dependent manner. HepG2 cells were treated with a series of concentrations (0, 0.25, 0.5, and 1 mM) of hydrogen proxide (H_2_O_2_) for 8, 16, or 24 hours. Relative HBA1 mRNA levels were determined by qRT-PCR. Data were expressed as Mean ± SD (n = 3). HBA1 level in control (0mM, 8hr) was normalized to 1. One way ANOVA and Tukey post hoc tests for multiple comparisons were used for analyzing the differences among treatments with different doses. **: p<0.01; ***: p<0.001, when compared to 0 mM treatment. **B**, HBA1 proteins were induced by H_2_O_2_ treatment. Whole cell lysates from HepG2 cells treated with or without H_2_O_2_ (1 mM, 48 hours) and blood cells were analyzed for HBA1 protein. Actin was probed as a loading control. **C**, HBB mRNA was upregulated by H_2_O_2_ treatment. HepG2 cells were treated with H_2_O_2_ (1 mM) for 48 hours. Reverse transcription PCR products were separated on 2% agrose gel and visualized with ethidium bromide. **D**, HBA1 and HBB mRNA in HEK293 cells were induced by H_2_O_2_ treatment. HEK293 cells were treated with H_2_O_2_ (1 mM) for 24 hours and hemoglobin mRNA levels were determined by qRT-PCR. Hemoglobin levels in controls were normalized to 1. Data were expressed as Mean ± SD (n = 3).

Oxidative stress is known to be elevated in NASH and, as a result, various stress responsive genes are up or down regulated. It is of interest to test whether hemoglobin expression can be regulated by oxidative stress. HepG2 cells were treated with hydrogen peroxide, a commonly used inducer for oxidative stress, and harvested for mRNA analysis and protein levels of hemoglobin. As shown in Fig. 4A, HBA1 mRNA levels were increased by H2O2 in a dose dependent manner at each time point tested. Consistently, HBA1 protein was also increased by H2O2 treatment as shown in Fig. 4B. Although HBB protein was undetectable in HepG2 cells, HBB mRNA was also induced by H2O2 treatment (Fig. 4C). Further, we tested if oxidative stress can induce hemoglobin expression in other non-erythrocytes. As shown in Fig. 4D, hemoglobin mRNA was also induced by H2O2 in human embryonic kidney HEK293 cells. In addition, we tested if oxidative stress has any effects on the expression of GATA1, a transcriptional factor regulating hemoglobin expression during erythropoiesis. As shown in Fig. S1, H2O2 significantly decreased the GATA1 expression.

### Reduction of oxidative stress by hemoglobin overexpression

**Figure 5:**
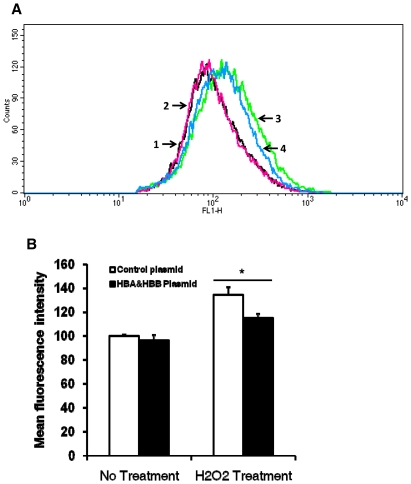
Reduction of oxidative stress by hemoglobin overexpression. HepG2 cells were transfected with control or HBA&HBB expression plasmid for 48 hours and then treated with H_2_O_2_ (1 mM, 5 minutes) or mock treated. Intracellular H_2_O_2_ levels were measured by flow cytometry. **A**, Representative histogram plots of different treatments. Plots 1 and 2: control and HBA&HBB plasmid without H_2_O_2_ treatment, respectively. Plots 3 and 4: control and HBA&HBB plasmid with H_2_O_2_ treatment, respectively. **B**, Quantitative analysis of mean fluorescence intensity. Data were expressed as Mean ± SD (n = 4).

As shown in Fig. 4, hemoglobin expression is inducible by oxidative stress. Next we attempted to determine its function in non-erythrocytes. Some oxidative stress inducible genes such as heme oxygenase 1 (HMOX1) have anti-oxidative functions [37], so we tested if hemoglobin overexpression had any effects on oxidative stress. HepG2 cells were transfected with control or pIRES/HBA&HBB plasmid expressing HBA1 and HBB for 48 hours and then treated with H2O2, followed by measuring intracellular oxidative stress by flow cytometry. As shown in Fig. 5A, in H2O2 treated cells, the histogram plot of hemoglobin overexpression was shifted to the left, indicating reduction of intracellular oxidative stress. Quantitative analyses of mean fluorescence intensity showed a significant reduction of oxidative stress in H2O2 treated and hemoglobin overexpressed cells, compared to H2O2 treated control cells (Fig. 5B).

## Discussion

The long-standing perception that hemoglobin is specifically expressed in erythrocytes has been challenged by recent studies showing hemoglobin expression in non-erythrocytes including neurons [24]–[26], retinal cells [27], [28], alveolar cells [29]–[31], mesangial cells of the kidney [32], and macrophages [33]. Our results showed that hemoglobin expression was increased in NASH (Table 1). Because hemoglobin usually is specifically expressed in erythrocytes, increased hemoglobin in NASH could suggest erythropoiesis is induced in NASH livers. During erythropoiesis, in addition to hemoglobin, other erythrocyte specific genes such as delta aminolevulinate synthase 2 (ALAS2), glycophorin A (GYPA), α- and β-spectrin (SPTA, SPTB), and hematopoietic transcription factor GATA1 [38], [39] should also be upregulated. But those genes did not show significant increases in NASH livers. Thus, it is unlikely that the increased hemoglobin in NASH resulted from erythropoiesis and it raised the possibility that hemoglobin was expressed by hepatocytes. The expression of HBA1 and HBB in hepatocytes was confirmed on liver biopsies using immunofluorescence staining (Fig. 2). Hemoglobin expression in human hepatocellular carcinoma HepG2 cell line (Fig. 3), a commonly used cell line in liver research, was also observed. This is the first report that hemoglobin is expressed in hepatocytes.

A recent proteomic study has shown that free hemoglobin α and β subunits in serum were significantly increased from normal controls to steatosis and to NASH, suggesting free hemoglobin subunits in serum could be a biomarker for liver lesions [20]. In another population study, higher free serum hemoglobin has been associated with higher prevalence rates of NAFLD [21]. However, the source of free hemoglobin in serum was not identified. It was assumed that free hemoglobin in serum was due to oxidative stress induced hemolysis. Our studies showed that hemoglobin was expressed in hepatocytes and it was increased in NASH. This provides a possible explanation for free hemoglobin in serum; free hemoglobin in serum could have been synthesized in hepatocytes and then released into the circulatory system. Increased serum free hemoglobin in NASH could reflect the increased hemoglobin production in the liver.

Erythropoiesis is a complicate multistep process in which pluripotent hematopoietic stem cells differentiate into erythrocytes. Hemoglobin expression is well coordinated and tightly controlled at the late stage of erythropoiesis. Several transcription factors are involved in erythroid differentiation. GATA1 is required for hemoglobin transcription and is also upregulated at the late stage of erythropoiesis [38], [39]. How hemoglobin expression is regulated in non-erythrocytes is not fully understood. It has been shown that hypoxia upregulates the transcription of GATA1 in alveolar epithelial cells. As a result, hemoglobin transcription is also increased by hypoxia [29]. Lipopolysaccharide (LPS) and interferon (IFN) induce hemoglobin beta expression in mouse macrophages [33]. Oxidative stress is elevated in NASH and is known to regulate expression of various genes. Increased hemoglobin expression could be related to oxidative stress in NASH. Upregulation of hemoglobin expression by oxidative stress in HepG2 and HEK293 cells indicated that oxidative stress is an important regulator for hemoglobin expression in non-erythrocytes. Interestingly, our results also showed that oxidative stress significantly decreased GATA1 expression in HepG2 cells (Fig. S1), suggesting that, unlike hypoxia, oxidative stress induces hemoglobin expression via a different mechanism. Transcription factor Nrf2 is known to be a crucial mediator for oxidative stress induced gene expression [40]. Further studies are needed to determine if Nrf2 mediates oxidative stress induced hemoglobin expression.

The main function of hemoglobin in erythrocytes is to transport oxygen from the lung to the tissues and to transport carbon dioxide from the tissues to the lung. Its function in non-erythrocytes is not clear. It was speculated that the function of hemoglobin in neurons could be oxygen storage [26]. Hemoglobin overexpression in mouse dopaminergic cell line resulted in changes in the expression of various genes involved in oxygen homeostasis and mitochondrial oxidative phosphorylation [24]. Another study has shown that in mouse renal mesangial cell line, SV40-MES13, hemoglobin overexpression reduced oxidative stress, suggesting hemoglobin functions as an antioxidant [32]. Recently, hemoglobin has been shown to function as an antioxidative peroxidase that reduces hydrogen peroxide-induced oxidative stress [41]. Consistently, we also showed that hemoglobin overexpression could suppress hydrogen peroxide-induced oxidative stress in HepG2 cells. Together, our findings suggest that elevated oxidative stress in NASH could induce hemoglobin expression and suppression of oxidative stress by hemoglobin could be a mechanism to protect hepatocytes from oxidative damage.

## Materials and Methods

### Liver Biopsies

The human studies are approved by the Institutional Review Board (IRB) of the State University of New York at Buffalo. With prior written consent from parents, liver biopsies were obtained, from patients suspected of having NASH as part of regular medical care. Patients from 7 to 18 years of age signed an assent to the research. All NASH patients had normal apha-1-antitrypsin levels, none were phenotype ZZ, and PAS staining of the biopsies was negative. All had normal or low ferritin and iron levels and soluble transferring receptor results were not consistent with iron overload. None of the liver biopsies had histological evidence of iron. None had any identifiable infectious agent including Hepatitis A, B, and C. All had normal serum copper and ceruloplasmin and all had normal celiac screens.

### Cell Culture

Human hepatocellular carcinorma HepG2 and embryonic kidney HEK293 cell lines were obtained from the American Type Culture Collection and were maintained in Dulbecco's modified Eagle's medium (DMEM) supplemented with 10% fetal bovine serum (FBS) at 37°C under 5% CO2.

### Microarray Analysis

Recently published microarry data including 12 NASH and 5 control samples was analyzed to identify differentially expressed genes in NASH [42], [43]. Total RNA of non-NASH controls was purchased from Admet Technologies (Durham, NC). The characteristics of NASH and controls were shown in Table 2. The original dataset has been uploaded to Gene Expression Omnibus (GEO; website: http://www.ncbi.nlm.nih.gov/gds). GEO accession number for the dataset is GSE24807.

**Table 2:** Characteristics of NASH and Control Groups.

	NASH (N = 12)	Control (N = 5)
Sex	F3:M9	F1:M4
Age (years)	13.9±2.7 (9–19)	10.4±6.1 (5–19)
BMI	32.4±7.1 (22–45)	NA
IR (HOMA)	4.2±2.4 (1.0–8.4)	NA

BMI: Body Mass Index; IR: Insulin Resistance;

HOMA: Homeostasis Model Assessment; NA: Not available.

### Quantitative Real Time PCR (qRT-PCR)

Total RNA was extracted from liver biopsies or cells using the RNeasy Mini Kit from Qiagen. Five hundred nanograms of total RNA was reverse transcribed to cDNA in 20 µl reaction using the iScript cDNA Synthesis Kit from Bio-Rad. One µl of the resulting cDNA was used in a total volume of 50 µl of PCR reaction. Real time PCR was performed on MyiQ PCR Detection System (Bio-Rad) using SYBR Green Supermix reagents. Glyceraldehyde-3-phosphate dehydrogenase (GAPDH) was used as internal control. Relative gene expression was calculated as previously described [42], [43]. The following primers were used for qRT-PCR.

GAPDH forward: 5′ AGCCTCAAGATCATCAGCAATG 3′;

GAPDH reverse: 5′ ATGGACTGTGGTCATGAGTCCTT 3′;

HBA1 forward: 5′ ACCCGGTCAACTTCAAGC 3′;

HBA1 reverse: 5′ AACGGTATTTGGAGGTCAGC 3′;

HBB forward: 5′ CTCGGTGCCTTTAGTGATGG 3′;

HBB reverse: 5′ ACACAGACCAGCACGTTG 3′.

### Immunofluorescence Staining

Liver biopsies were fixed in 4% formaldehyde for 15 minutes at room temperature and then washed twice with TBS. Fixed biopsies were transferred to 30% sucrose and kept at 4°C overnight. Biopsies were then embedded in OCT compound in cryomolds. Twenty micro meters of cryosections were made on a cryostat machine and stored at −80°C until staining. Frozen sections were incubated in PBS for 15 minutes, followed by permeabilization with 0.75% triton X-100 for 15 minutes and antigen retrieval in 1% sodium dodecyl sulfate (SDS) for 5 minutes. Sections were incubated with HBA1, HBB, or radixin antibody (5 ng/µl) for 90 minutes and then blocked with 4% BSA for 30 minutes. Second antibody conjugated with Alexa 568 or Alexa 488 (2 ng/µl) was incubated with the sections for 45 minutes. Coverslips were mounted onto sections using mounting reagent containing 4,6-diamidino-2-phenylindole (DAPI). Immunofluorescence images were recorded under LSM510 beta laser scanning microscope (Zeiss) using same instrument settings for all samples.

### Plasmid Transfection

HepG2 cells were transfected with a control plasmid or pIRES/HBA&HBB [32] (Kind gift from Dr. Masaomi Nangaku, University of Tokyo School of Medicine) using GenJet transfection reagents (SignaGen), according to manufacturer's instructions. Forty eight hours after transfection, cells were harvested for western blot analysis or used for flow cytometry.

### Western Blot Analysis

Whole cell lysates from HepG2 cells were made in SDS lysis buffer. Protein concentrations were measured using a modified Bradford method [44]. Sixty µg of whole cell lysates were separated by 15% SDS polyacrylamide gel electrophoresis and transferred onto nitrocellulose membrane. After blocking with 5% milk for 30 min, the membrane was incubated with primary antibody for 2 hours and then washed three times, followed by incubation with horseradish peroxidase-conjugated secondary antibody for 1 hour. After washing three times, the protein bands were visualized using ECL reagents (Pierce). The images were recorded with a Fuji Image Reader LAS-1000. HBA1 and HBB antibodies were purchased from Santa Cruz. Beta actin antibody was obtained from MP Biomedicals.

### Detection of oxidative stress by flow cytometry

Oxidative stress was measured according to a published method [32]. Briefly, HepG2 cells were washed with PBS and then incubated with 10 µM of 5-(and-6)-chloromethyl-2′,7′-dichlorodihydroflurescein diacetate, acetyl ester (CM-H2DCFDA; Invitrogen) in the dark for 30 minutes at 37°C. After washing with PBS, HepG2 cells were treated with 1 mM H2O2 for 5 min or left without treatment, followed by harvesting the cells by trypsin. The CM-H2DCFDA fluorescence signals were collected immediately in the FL-1 (530 nm) channel on a FACScalibur machine (Becton Dickinson). Mean fluorescence intensity (MFI) was analyzed by the CELL Quest Pro software.

### Statistic Analysis

Unpaired student t tests were performed to analyze the differences between control and experiment groups. One way ANOVA and Tukey post hoc tests for multiple comparisons were used for analyzing the differences among H2O2 treatments with different doses. A p value less than 0.05 was considered to be statistically significant.

## Supporting Information

Figure S1 Down regulation of GATA1 expression by oxidative stress. Control (0 mM) and H2O2 treated (1 mM) samples were same as those used in Figure 4A. Relative GATA1 mRNA levels were determined by RT-PCR. Data represents Mean ± SD for three RT-PCR reactions. Compared to control, H2O2 treatment for 8, 16, or 24 hours significantly decreased the GATA1 mRNA levels (t test, p<0.05).
(TIF)
