# Supplementary figures and images for: Upregulation of Hemoglobin Expression by Oxidative Stress in Hepatocytes and Its Implication in Nonalcoholic Steatohepatitis
Source: PLoS One. 2011 Sep 12;6(9):e24363. doi: 10.1371/journal.pone.0024363 (PMC3171444; doi:10.1371/journal.pone.0024363)

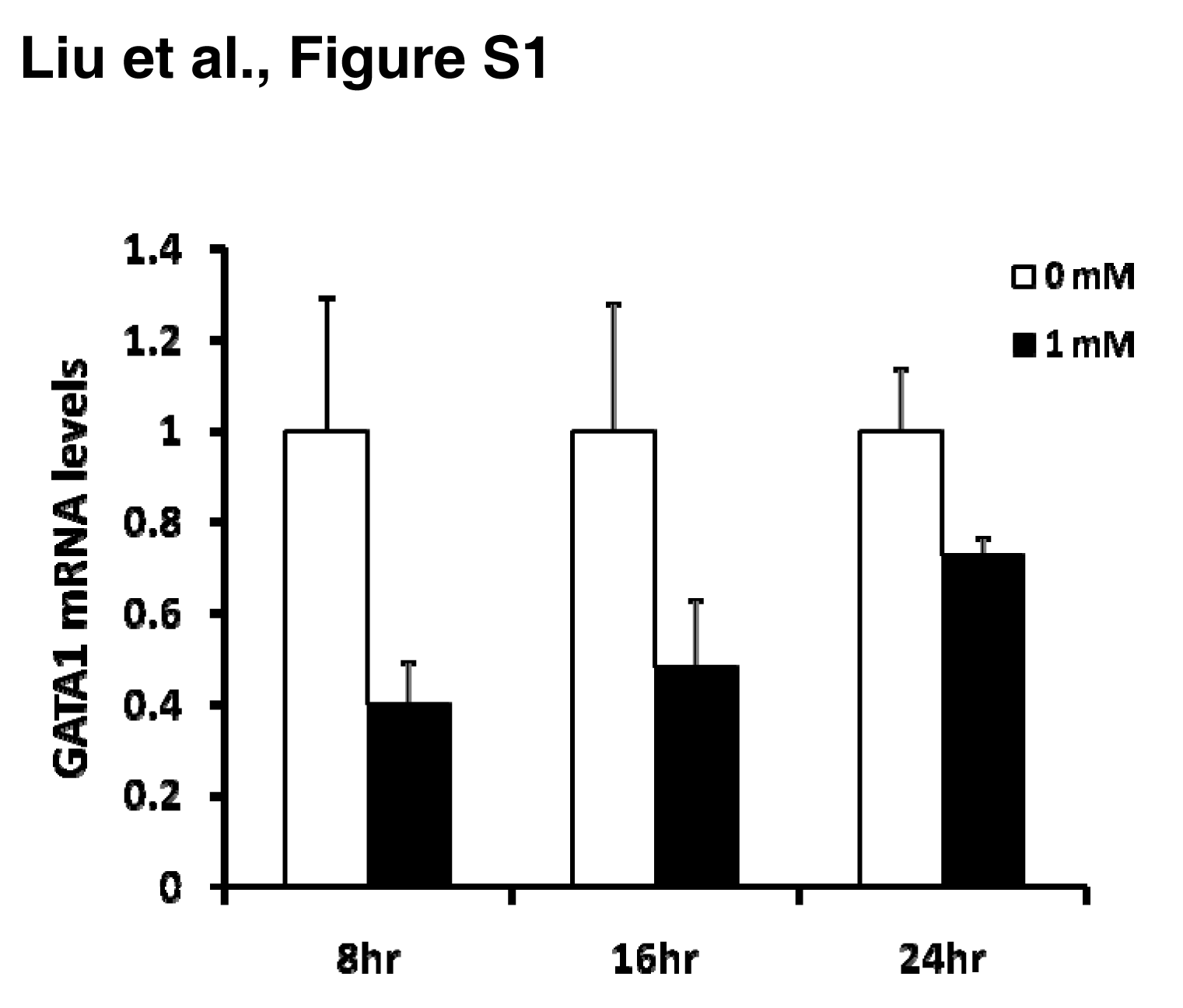

Supplement: Figure S1 — Down regulation of GATA1 expression by oxidative stress. Control (0 mM) and H2O2 treated (1 mM) samples were same as those used in Figure 4A. Relative GATA1 mRNA levels were determined by RT-PCR. Data represents Mean ± SD for three RT-PCR reactions. Compared to control, H2O2 treatment for 8, 16, or 24 hours significantly decreased the GATA1 mRNA levels (t test, p<0.05). (TIF) [file pone.0024363.s001.tif]
